# Evaluation of Facial Asymmetry in Patients with Crooked Nose

**DOI:** 10.1007/s00266-024-04235-3

**Published:** 2024-07-15

**Authors:** Ergin Eroğlu, A. Erim Pamuk, Ömer Taşkın Yücel

**Affiliations:** Department of Otorhinolaryngology, Hacettepe University, Ankara, Turkey

**Keywords:** Crooked nose, Facial asymmetry, Cone beam computed tomography

## Abstract

**Background:**

This study aims to explore the correlation between facial asymmetry and a crooked nose using objective methodologies.

**Methods:**

The cohort of 57 patients who underwent septorhinoplasty surgery for aesthetic reasons between 2019 and 2022. Patients were categorized based on the type of nasal axis deviation. The analysis involved reviewing patients’ photographs and cone beam computed tomography images. We identified various anatomical landmarks and compared measurements across the groups.

**Results:**

Among the study population, 21 (36.8%) exhibited Type-I (linear) and 15 (26.3%) demonstrated Type-C nasal axis deviation, while no deviation was detected in 21 (36.8%) patients. Upon evaluating the upper face area, significant differences were found in the glabella-lateral orbit (G-LO) and rhinion-lateral orbit (Rh-LO) parameters (*p *= 0.002 and *p *< 0.001, respectively). A statistically significant difference was discovered in all three parameters between the three groups in the middle face area [glabella-zygion (G-Zy) *p *= 0.04, rhinion-zygion (Rh-Zy) *P *< 0.001, anterior nasal spine-zygion (ANS-Zy) *p *< 0.001)]. Further, a statistically significant difference was noted in the soft tissue parameters gonion (Go) and LO (*p *= 0.008 and *P *= 0.005, respectively).

**Conclusion:**

Patients with crooked noses, in particular, exhibit asymmetries in the upper and middle faces. The glabella in the upper face and the anterior nasal spine in the middle face are stable points, and the fact that the parameters derived from these two reference points are significant, when considered in conjunction with other significant parameters, strongly supports the aforementioned statement.

**Level of Evidence III:**

This journal requires that authors assign a level of evidence to each article. For a full description of these Evidence-Based Medicine ratings, please refer to the Table of Contents or the online Instructions to Authors www.springer.com/00266.

## Introductıon

Septorhinoplasty, typically conducted for aesthetic purposes, is a commonly performed surgical procedure for both cosmetic and functional benefits [1]. This surgery prioritizes patient satisfaction, with particular emphasis on patients with crooked noses. These patients necessitate thorough evaluation and meticulous surgical planning to ensure satisfactory outcomes [2].

The term ‘crooked nose deformity’ describes a nose that deviates to one side relative to the face. This deformity is further subdivided into three types: Type-I (linear), Type-C, and Type-S. Type-I nose deviation involves a linear shift of the nose to one side away from the vertical midfacial line [3]. Type-C nose deviation signifies a shift to one side of the vertical midfacial line, imparting a concave or convex appearance [4]. Type-S denotes a complex deviation with the dorsum and tip of the nose in disparate locations [3, 5].

Asymmetry refers to a difference in size and shape between the two sides of the face within the craniofacial area. This difference could stem from hard tissues or be confined to soft tissues [6]. A multitude of studies have explored the relationship between facial asymmetry and a crooked nose [7, 8]. A study by Nouraei et al. [6] reported significant facial asymmetry in 97% of septorhinoplasty candidates. Additionally, patients with a crooked nose may sometimes complain of residual nasal axis deviation post-surgery, oblivious of the underlying facial asymmetry [9]. Hence, it is essential to consider the possibility of facial asymmetry in patients with a crooked nose. Symmetrical facial features contribute to the perception of attractiveness [10]. Therefore, objectively elucidating the relationship between facial asymmetry and a crooked nose and communicating potential postoperative scenarios are crucial for both patient and surgeon satisfaction.

Our hypothesis posits that patients with a crooked nose exhibit facial asymmetry. The objective of our study is to scrutinize the relationship between facial asymmetry and a crooked nose using objective methodologies.

## Materials and Methods

Ethical approval was procured from the Hacettepe University Committee of Ethics (No. GO/21-2123). Patients seeking septorhinoplasty surgery for aesthetic concerns who presented at Hacettepe University, Department of Otorhinolaryngology, were screened between January 1, 2019, and December 31, 2022. Informed contest was taken from the patients.

The study excluded patients who had previously undergone nasal or maxillofacial surgery, experienced maxillofacial trauma, possessed craniofacial anomalies or jaw asymmetry, had temporomandibular joint ankylosis or dislocation, lacked sufficient image quality, had traumatic deviations, or were outside the age range of 18–65.

Both internal and external nasal examination findings were noted. This included potential septum deviation via anterior rhinoscopy and endoscopic nasal examination, the type of deviation (cartilage, bone, or combined), presence of bony spur in the posterior, concha hypertrophy, and the condition of the nasal mucosa. The external nasal examination took note of the radix and nasal dorsum condition (presence of a nasal hump and its origin bone, cartilage, or combined structures), the location of the nasal tip point, the relationship between the dorsum and the nasal tip, and the type of nose deviation (I, C, or S type), if any, when viewed from the profile.

To ensure standardization, photographs were taken by a single individual. Extraoral photographs were captured from an average distance of 1.5 m using a Canon EOS 2000D. Patients were positioned freely, with the Frankfurt horizontal plane parallel to the ground.

The images evaluated in the study were sourced from the i-CAT Next Generation® (Imaging Sciences International, Hatfield, Philadelphia, USA), and the RadiAnt Dicom Viewer® (Medixant, Poznan, Poland) was employed for image analysis. Cone beam computed tomography (CBCT) images were used in Digital Imaging and Communications in Medicine (DICOM) format.

**Fig. 1:**
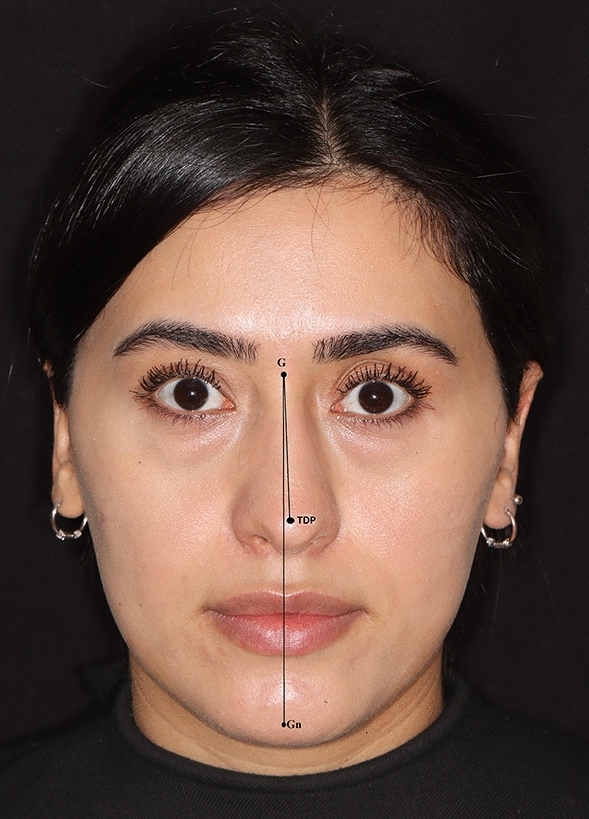
Type-I nasal axis deviation. G: glabella, TDP: tip defining point, Gn: gnathion

**Fig. 2:**
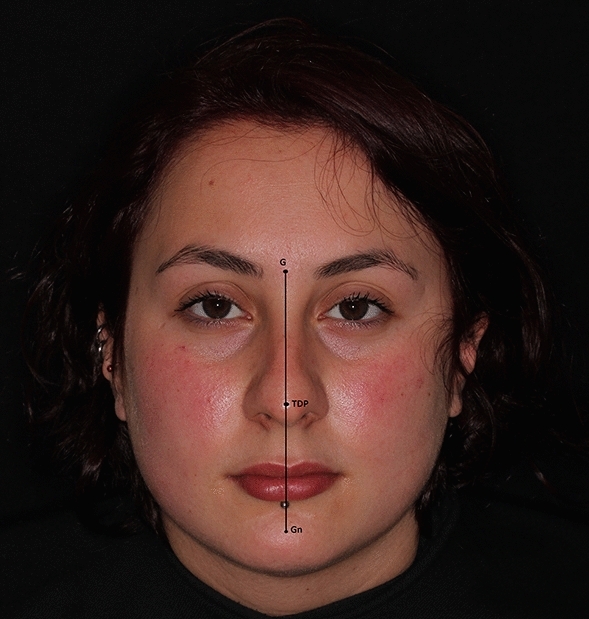
Normal patient without nasal axis deviation. G: glabella, TDP: tip defining point, Gn: gnathion

For the identification of Type-I (linear) nasal axis deviation, key anatomical reference points such as the glabella, rhinion, and tip defining point (TDP) were located and interconnected using a linear vector. Subsequently, the gnathion was marked and linked to the glabella utilizing an alternate linear vector. A Type-I (linear) nasal axis deviation was deemed present if these two vectors did not align on a single plane (Fig. 1). Conversely, if the vectors were situated on the same plane, it was inferred that no nasal axis deviation existed, thus forming a control group (Fig. 2). Additionally, the angle formed by the intersection of these two vectors was calculated.

**Fig. 3:**
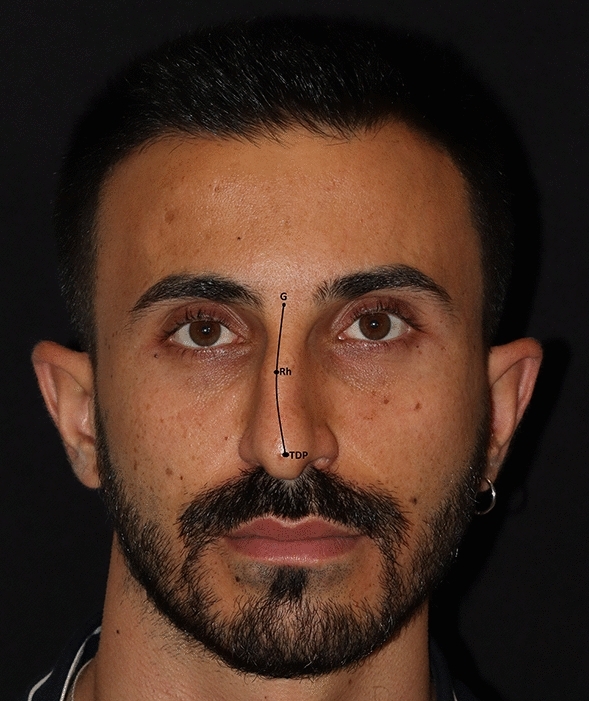
Type-C nasal axis deviation. G: glabella, Rh: rhinion, TDP: tip defining point

For the identification of Type-C nasal axis deviation, the same initial anatomical landmarks (glabella, rhinion, and tip defining point (TDP)) were pinpointed. If these three points did not reside on a single plane, they were interconnected using a concave angular vector (Fig. 3).

**Fig. 4:**
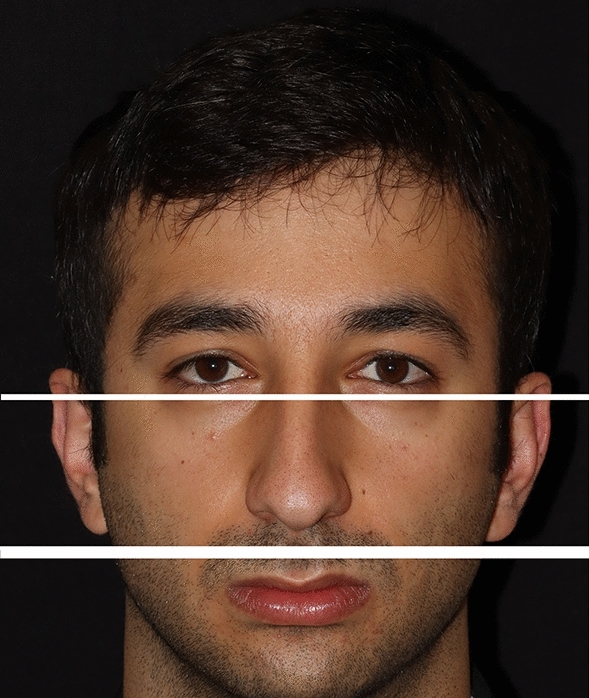
Photographic depiction of the upper, middle, and lower facial regions

All data were individually assessed for the right and left sides of the face. Prior to delineating the process for data measurement management, the photographs of the patients were segregated into three distinct regions: the upper, middle, and lower face (Fig. 4). The parameters delineated in the subsequent section were devised to elucidate the interrelationships within and between these three facial areas.

Upon completion of soft tissue measurements within both the axial and coronal planes, a new image window was initiated using 3D reformat for the execution of bone distance measurements. The processing of all image sequences commenced, with measurements initiated by identifying the control points (Table 1).

**Table 1:** Measurement parameters of control points

G-LO	Glabella-Lateral Orbit
Rh-LO	Rhinion-Lateral Orbit
G-Zy	Glabella-Zygion
Rh-Zy	Rhinion-Zygion
ANS-Zy	Anterior nasal spine-Zygion
G-AM	Glabella-Angle of mandible
Rh-AM	Rhinion-Angle of mandible
Gn-AM	Gnathion-Angle of mandible
Ch-LO	Chellion-Lateral Orbit
Ch-Go	Chellion-Gonion

**Fig. 5:**
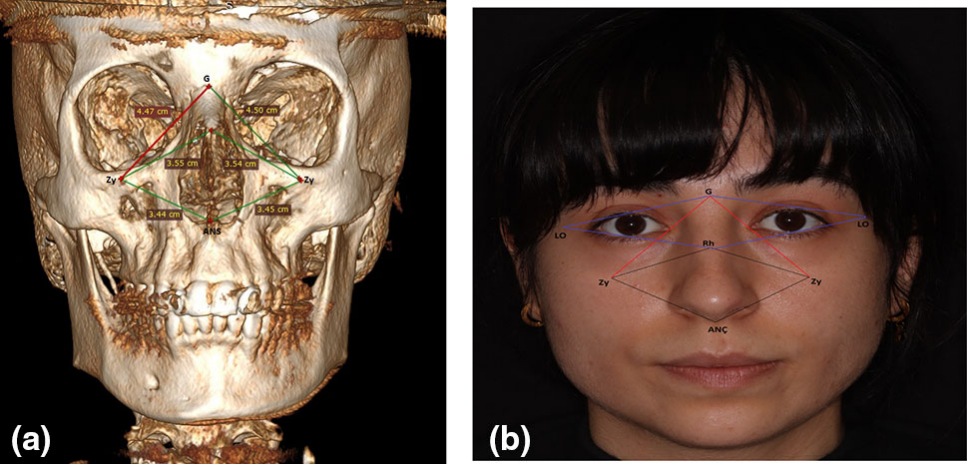
The measurement of distances between Zy-ANS, Z-Rh, Zy-G, G-LO, and Rh-LO on both sides of the face were depicted through 3D CT (**a**) and photography (**b**). Zy: Zygion, ANS: Anterior Nasal Spine, Rh: Rhinion, G: Glabella, LO: Lateral Orbit

Initially, in the analysis of the middle face, three distinct measurements were computed based on the zygion (Zy). Distances from the most prominent point beneath the inferior wall of the orbit, coinciding with the zygomaticomaxillary suture line (Zy), to the anterior nasal spine (ANS), rhinion (Rh), and glabella (G)—the junction of the nasal and frontal bones—were ascertained. Subsequently, two measurements were conducted based on the lateral orbit (LO) to evaluate the upper facial region. The inner edge of the frontozygomatic suture line served as the reference for LO, and the distances to the glabella (G) and rhinion (Rh) were independently measured (Fig. 5a, b). Additionally, to examine the image from a more lateral perspective, the distance at the Rh region was gauged after applying a rotation, and the nasal bone (NB) length in this region was documented for both the right and left sides.

**Fig. 6:**
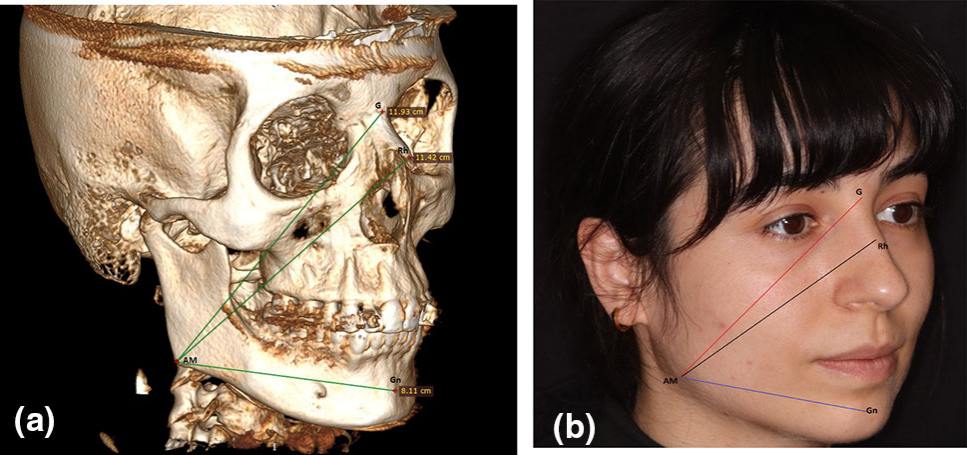
The measurement of distances between G-AM, Rh-AM, and Gn-AM on right side of the face were depicted through 3D CT (**a**) and photography (**b**). G: Glabella, Rh: Rhinion, AM: Angulus Mandibula, Gn: Gnathion

The calculation of the face’s vertical length involved measuring the distances between the angle of the mandible (AM) and both G and Rh. Moreover, the distance between AM and gnathion (Gn), deemed as the midpoint of the chin, was also measured to gather data pertinent to the lower facial region (Fig. 6a, b).

**Fig. 7:**
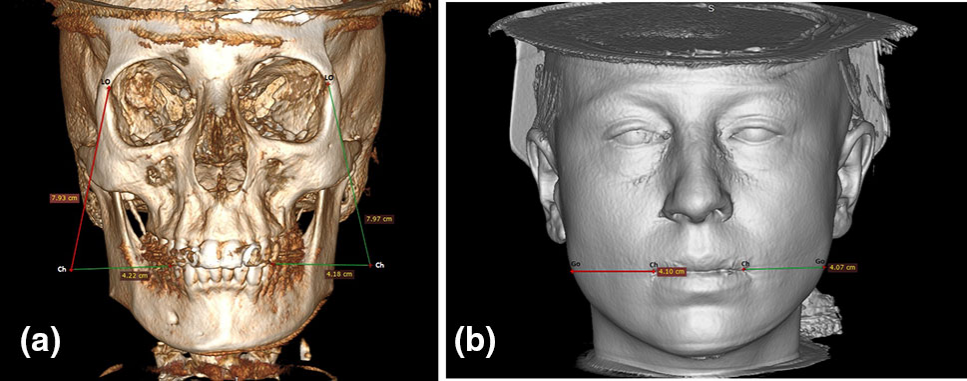
The measurement of distances between Ch-Go and LO-Ch on both sides of the face were depicted through 3D CT skin format (**a**) and 3D CT bone format (**b**). Ch: Chellion, Go: Gonion, LO: Lateral orbit

A new image template was generated by overlaying the subcutaneous and skin layers onto the bone image procured via 3D reformatting. On this template, the distance between the oral commissure, also termed chellion (Ch), and the gonion was measured bilaterally (Fig. 7a, b). Subsequently, data measurement was concluded by quantifying the distance between Go and LO, marked on the same image after reverting to the bone format.

The data analysis was conducted using the SPSS 25.0 program at a 95% confidence level. The Chi-square, Mann–Whitney, Kruskal–Wallis, dependent group t-test, Pearson, and Spearman correlation tests were utilized. The relationship between categorical variables was analyzed with the Chi-square test. For parameters that are not normally distributed, the comparison of measurements regarding two-group variables was conducted with the Mann–Whitney test, and for three or more groups, the Kruskal–Wallis test was employed. For parameters that followed a normal distribution, the ANOVA test was used. In the correlation analysis performed between the angles obtained from the axis deviation and the measurement parameters, the Spearman correlation test was used for the non-normally distributed group, and the Pearson correlation test was utilized for the normally distributed group.

## Results

Among the 57 participants in the study, 32 (56.1%) were women and 25 (43.9%) were men. Fifty (87.7%) patients reported nasal congestion, and septal deviation was identified in 44 of them (77.1%). Septal deviation was also detected in 2 of the 7 patients (3.5%) who did not report nasal congestion. Table 2 shows the demographic and clinical characteristics of the study group.

**Table 2:** Demographic and clinical features of the study cohort

Features	Number = 57, (%)
Age, Mean ± standard deviation, year	25.54 ± 6.21
Male/Female	25/32 (43.9/56.1)
Nasal congestion	50 (87.7)
Septal deviation	46 (80.7)
*Deviation type*	
Anterior right deviation	19 (33.3)
Anterior left deviation	19 (33.3)
Isolated bone spur	1 (1.8)
Mix	8 (14)
*Nasal axis deviation*	
Normal	21 (36.8)
Type-I	21 (36.8)
Type-C	15 (26.3)

The initial physical examination and subjective evaluation identified that 22 patients (38.6%) exhibited Type-I, 12 (21.1%) exhibited Type-C deviation, and 23 (40.3%) displayed no nasal axis deviation. Subsequently, a photographic analysis was performed to create the three groups for our study. The objective photographic evaluation revealed that 21 (36.8%) patients had Type-I, 15 (26.3%) had Type-C nose deviation, and 21 (36.8%) patients had no nasal axis deviation, as illustrated in Table 2.

Photographic analysis of the initial 22 patients, classified via physical examination as having Type-I nasal axis deviation, indicated that merely 14 (63.6%) of them exhibited Type-I deviation. Conversely, 3 (13.6%) demonstrated Type-C, and 5 (22.7%) showed no deviation. Furthermore, among the 23 patients initially perceived as having no deviation, 5 (21.7%) exhibited Type-I, and 2 (8.7%) demonstrated Type-C nasal axis deviation.

A total of 11 distinct parameters were evaluated using CBCT. Their mean values, along with an analysis based on the type of deviation, are illustrated in Table 3.

**Table 3:** Mean values of CBCT measurement parameters and results of intergroup statistical analysis results

	Relationship	Normal (mean±SS)	Type-I (mean±SS)	Type-C (mean±SS)	*p*	Subgroups	*p*
G-LO	Upper face	0.9840±0.01232	0.9586±0.04638	0.9295 ±0.05794	0.002*	N-I N-C I-C	0.054 **0.01γ** 0.88
Rh-LO	Upper face	0.9828±0.01496	0.9411±0.04762	0.9212±0.04881	<0.001*	**N-I** **N-C** I-C	**0.003 γ** **<0.001γ** 0.3
G-Zy	Middle face	0.9722±0.03911	0.9709±0.02528	0.9554±0.02669	0.04*	N-I **N-C** I-C	0.641 **0.011 γ** 0.069
Rh-Zy	Middle face	0.9752±0.02139	0.9355±0.05052	0.8724±0.09364	<0.001*	N-I **N-C** I-C	0.02 **<0.001γ** 0,043
ANS-Zy	Middle face	0.9816±0.02322	0.9390±0.05024	0.9125±0.0760	<0.001*	**N-I** **N-C** I-C	**0.01 γ** **<0.001γ** 0.289
Nasal bones	Upper face Middle face	0.9514±0.05683	0.8497±0.10330	0.8092±0.10418	<0.001*	**N-I** **N-C** I-C	**0.001 γ** **<0.001γ** 0.379
G-AM	Vertical symmetry	0.9827±0.02442	0.9308±0.18938	0.9732±0.02559	0.179	N-I N-C I-C	0.056 0.309 0.687
Rh-AM	Vertical symmetry	0.9801±0.02041	0.9666±0.03757	0.9635±0.03401	0.316	N-I N-C I-C	0.313 0.130 0.688
Gn-AM	Lower face	0.9806±0.02399	0.9629±0.02363	0.9658±0.02655	0.056	N-I N-C I-C	0.61 0.263 0.983
Ch-Go	Lower face	0.9629±0.03144	0.9321±0.05024	0.9368±0.04909	0.09	N-I N-C I-C	0.056 0.86 0.069
LO-Ch	Vertical symmetry	0.9787±0.02097	0.9657±0.02927	0.9583±0.02870	0.068	N-I N-C I-C	0.285 0.081 0.840

*G* glabella, *LO* lateral orbit, *Rh* rhinion, *Zy* zygion, *ANS* anterior nasal spine, *AM* angle of mandible, *Gn* gnathion, *Ch* chellion

*Statistical significance for *p* < 0.05, Bold indicates statistical significance

^γ^Statistical significance for *p* < 0.016, Bold indicates statistical significance

Alterations in angle values in those presenting with Type-I and Type-C deviations underscore the prominence of the curved nose deformity. Notably, no significant correlation, whether positive or negative, was detected between the angle values and any of the measurement parameters within both the Type-I and Type-C groups. Furthermore, there was no significant association between septal deviation and any of the measured parameters.

## Discussion

Patients with crooked nose deformity often present a challenge to even the most experienced surgeons. During an initial patient encounter, the relationship of the nose to the midline is a primary point of observation. Any discrepancies or irregularities in the alignment, orientation, and relation of the nasal pyramid and the cartilaginous framework to the face as a whole can immediately stand out. However, certain asymmetries and crooked nose deformities may go unnoticed if not meticulously examined. For instance, in our study, photo analysis revealed Type-I deviation in 5 (21.7%) and Type-C deviation in 2 (8.7%) of the 23 patients initially considered to have a normal nose upon inspection. Additionally, 3 (13.6%) of the 22 patients initially diagnosed with Type-I nose deviation based on a physical examination were later found to have Type-C deviation.

There are various methods to evaluate crooked nose deformity. Jong and colleagues have identified five different conditions based on the positions of the nasal pyramid and the cartilaginous framework [11]. Type-I, Type-C, and Type-S deviations’ classification being widely cited in the literature [5, 12, 13]; as with our study, no patients with Type-S deviations were identified, likely attributable to the rarity of this specific anomaly.

### Relationship Between Nasal Septum Deviation and Facial Asymmetry

In our cohort, 50 out of 57 patients (87.7%) had nasal congestion, with septal deviation identified in 46 patients (80.7%). The bulk of septal deviations manifest as right or left deviations situated in the cartilaginous part. A study by Kim et al. found right septal deviation in 13 out of 25 patients (52%) and left septal deviation in 12 (48%) [14]. The study further reported a statistically significant association between the discrepancy in the middle sagittal plane—zygion, glabella—exocanthion, cheilion—zygion between the right and left face, and the direction of septal deviation. Nevertheless, our study found no significant association between facial asymmetry and the direction or varied states of septal deviation. Though numerous studies propose that septum deviation, resulting from trauma or surgery early in life, can cause facial growth differences and asymmetry [15, 16], contrary findings have also been reported [17]. Our perspective is that the nasal septum alone is not a determinant of facial asymmetry; we posit that its association and positioning relative to other structures forming the nose are of greater significance.

### The Application of CBCT in Facial Asymmetry

Facial symmetry has been a long-standing subject of study in daily practice. However, there is presently no universally accepted method for scientifically revealing facial symmetry or asymmetry. Whereas many previous studies have employed cephalometric measurement methods, others have utilized computed tomography. CBCT, frequently used in dentistry, provides significant advantages such as reduced radiation exposure and cost-effectiveness. Distinct from the cephalometric method, 3D reconstruction has carved a new path in evaluating facial asymmetry by enabling the assessment of both soft and hard tissue [18, 19]. Recent studies also recommend CBCT for evaluating facial asymmetry and understanding patient facial morphology using 3D imaging [20, 21]. Nevertheless, the use of low-dose CBCT can limit the visibility of artifacts caused by implants and filling materials. In our study, due to the low dose, sufficient image quality could not be achieved in some patients, especially in the evaluation of the rhinion region, and these patients were excluded from the study.

The selection of reference points on the face’s midline also poses a challenge. With 3D reconstruction, one can eliminate soft tissues and access various landmarks on the skeleton. Particularly in determining the main reference points, different studies have used varying planes and points. Swennen and colleagues [22] advocate for the sella—nasion—menton line as the midline, while Cho and colleagues [23] have favored the sella—nasion—basion line. Another study considered the nasion—anterior nasal spine and posterior nasal spine line as the closest to the morphometric symmetric plane of the face [24]. Based on these studies and other literature information, we formed our measurement planes by referencing the glabella, anterior nasal spine, and gnathion landmarks located on the anterior midline of the face. Although the gnathion sometimes exhibits asymmetric positioning in patients with jaw asymmetry, our study included no patients with jaw asymmetry, temporomandibular joint ankylosis, or dislocation. Even though the anterior nasal spine, which we accepted as the midline landmark in the midface, is reported to be fractured with a nasal fracture in some publications [25, 26], none of the patients participating in the study had a history of major trauma.

### Parameters Utilized in Assessing Facial Asymmetry and Their Evaluation

Upon reviewing the existing literature, we found that various studies employed diverse reference points and planes to investigate facial asymmetry. In research conducted by Hafezi et al. [7], a statistically significant variance was identified in the asymmetrical face group regarding measurements between chellion—lateral orbita and rhinion—zygion points. In the evaluation of bilateral facial harmony, the Rh-Zy metric was deemed especially significant. In our study, although the Rh-Zy metric yielded significant results, the Ch-LO measurement did not.

In the study carried out by Dasdar et al. [13], 19 patients had Type-I deviation and 44 patients had Type-C deviation. A subjective analysis of the photos revealed that while 22 patients (34.9%) did not show any asymmetry, facial asymmetry was detected in 41 patients (65.1%). Statistically significant differences were observed in the distances between the midline-lateral canthus for the upper face, the midline—tragus for the middle face, and the midline—oral commissure for the lower face. Our study also derived significant results for G-LO for the upper face, ANS-Zy for the middle face, and Rh-Zy, thereby corroborating the literature. A discernible difference was found between the patient group with a specific Type-C deformity and the control group in a subgroup analysis. In addition, the fact that the Rh-LO and G-Zy parameters also yielded significant results may suggest that the upper and middle face’s development are dynamic structures that influence each other. Furthermore, the nasal bones yielding statistically significant results in both the N-C and N-I groups underscores the difference in nasal bone lengths in patients with crooked noses. The foundation of the deformity in patients with crooked noses resides in the opening angles of the maxilla with the nasal bones and the disparity in the length of the nasal bones. Numerous studies in the literature investigate nasal deviation and facial asymmetry [27–29]. It is important to remember that concentrating exclusively on nose asymmetry may overlook asymmetry in other facial areas and their interrelations, potentially leading to patient dissatisfaction in the future.

A total of 85 different planes, created from the reference points on the midline and both sides of the face—considered reference planes on the CBCT over the nasion—anterior nasal spine and posterior nasal spine—were compared in groups with and without facial asymmetry [24]. The group deemed to have an asymmetrical face exhibited higher values in 77 of the 85 planes. Moreover, a statistically significant difference was determined in 25 of the 50 planes (50%) produced from the midline reference points and in 27 of the 35 planes (77%) generated from bilateral planes between symmetric and asymmetric face groups.

To the best of our knowledge, our study is one of the most comprehensive studies to integrate patient photographs, CT images, and 3D reformats to provide an objective assessment of the relationship between facial asymmetry and nasal deviation. We considered the face in three dimensions, examining it as an integrated whole. In contrast, many previous studies have explored the relationship between facial asymmetry and nasal deviation using two-dimensional photographs or computed tomography (CT) scans [8, 30]. Unlike these studies, our research elucidated the association between specific landmarks in the upper and middle facial regions within the 3D plane and the crooked nose. It was determined that the lower facial region does not exhibit a relationship with the crooked nose. This observation is consistent with the embryological growth patterns that occur during the maturation of the nose and face to their adult morphology [31].

Our study could be further strengthened and enhanced through certain steps. The first of these is increasing our patient numbers, which were limited due to the COVID-19 pandemic. Alongside this, conducting measurements with two or more observers would lend additional credibility to our study’s findings. Moreover, by defining different reference points and novel parameters, the analysis pool could be expanded with additional variables, such as depth measurement of the face, and volume measurement.

## Conclusion

It is plausible that satisfactory results might not be obtained through examinations conducted with inspection, and photographs of patients taken with specific rules should be scrutinized in detail for the relationship between the nose axis and other structures. In our study, we concluded that there were significant asymmetries, especially in the upper and middle face, in patients with crooked noses. Particularly in the upper face, the glabella and in the middle face, the anterior nasal spine are stable points. The fact that the parameters derived from these two reference points are significant, when considered together with other significant parameters, strongly supports the above assertion. Lastly, it has been objectively demonstrated that the lengths of the nasal bones differ in patients with crooked nose.
